# Comparison of basal whole‐body protein kinetics and muscle protein synthesis between young and older adults

**DOI:** 10.14814/phy2.14633

**Published:** 2020-12-05

**Authors:** Katie R. Hirsch, David D. Church, Il‐Young Kim, Sanghee Park, Robert R. Wolfe, Arny A. Ferrando

**Affiliations:** ^1^ Department of Geriatrics Donald W. Reynolds Institute on Aging Center for Translational Research in Aging and Longevity University of Arkansas for Medical Sciences Little Rock AR USA; ^2^ Department of Molecular Medicine Lee Gil Ya Cancer and Diabetes Institute College of Medicine Gachon University Incheon South Korea

**Keywords:** aging, anabolic resistance, protein metabolism, sarcopenia, stable isotopes

## Abstract

Significant research has been dedicated to counteracting age‐related muscle loss, but underlying mechanisms have not been clearly established. Previous research examining differences in basal protein kinetics between young and older individuals has been limited by a lack of evaluation of protein breakdown and net balance. The aim of this study was to more comprehensively examine differences in basal protein kinetics between younger and older males and females. Basal whole‐body protein kinetics and muscle fractional synthetic rate (FSR) from 91 younger (18–38 years; 52% female) and 66 older (51–81 years; 53% female) healthy adults were determined using stable isotope tracer techniques (L‐[ring‐^2^H_5_]phenylalanine and L‐[ring‐^2^H_2_]tyrosine). There were no group × sex interaction effects (*p* > .05). Older individuals had greater whole‐body protein synthesis (mean difference old‐young (Δ) ± SE: 28.54 ± 8.15 mg/kg LBM/hr; *p* = .001) and breakdown (Δ: 15.44 ± 7.33 mg/kgLBM/hr; *p* = .038), but a less negative net balance (Mean ± *SD*: Young: −31.22 ± 7.42 mg/kg LBM/hr; Old: −18.11 ± 21.60 mg/kg LBM/hr; *p* < .001) compared to young individuals. Basal FSR was not significantly different between young and older (Δ: 0.007 ± 0.003%/hr; *p* = .052). Across the age range, females had greater whole‐body protein turnover (PSΔ: 19.10 ± 7.00 mg/kgLBM/hr; PBΔ: 19.22 ± 6.31 mg/kgLBM/hr; *p* < .01) compared to males. Results demonstrate a difference in basal whole‐body protein kinetics between young and older adults, with older adults having a higher protein turnover rate and a less negative net balance. Across the age range, females were also found to have a higher turnover rate compared to males. Differences may represent a shift in older physiology toward mechanisms that increase the efficiency of amino acid reutilization, especially in women.

## Graphical abstract

Results demonstrate a difference in basal whole‐body protein kinetics between young and older adults, with older adults having a higher protein turnover rate and a less negative net balance. Women were also found to have higher protein turnover than men, regardless of age. Differences may represent a shift in physiology toward mechanisms that increase the efficiency of amino acid reutilization.

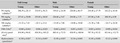

## 1 INTRODUCTION

Beginning at approximately 30 years of age, relative muscle size and strength begin to decrease until the age of 50, at which point the rate of decline accelerates, resulting in a loss of 0.5%–1% per year (Janssen et al., 1985; Mitchell et al., 2012). Age‐related decline in muscle size and quality is associated with increased risk of metabolic disease, decreased physical function, increased fall risk, and lower overall quality of life (Tieland et al., (2018); Wolfe, 2006). Much research has been dedicated to counteracting age‐related muscle loss, but the underlying mechanisms of sarcopenia have not been clearly established.

Suggested theories for age‐related muscle loss include DNA damage, reduced protein synthesis, fiber type changes, physical inactivity, inadequate nutrition/protein intake, and hormonal changes (Doherty, 2001; Tieland et al., 2018). Significant attention has been directed at characterizing and counteracting anabolic‐resistance, or the reduced anabolic response to protein intake that occurs with aging (Burd et al., 2013; Phillips et al., 1985). Differences in basal protein kinetics have been less rigorously evaluated. Previous work in this area has generally shown minimal differences in basal muscle protein synthesis between young and older individuals (Cuthbertson et al., 2005; Markofski et al., 2015; Moore et al., 2015; Volpi et al., 2001), with a few studies suggesting lower synthesis rates in older individuals (Balagopal et al., 1997; Welle et al., 1993; Yarasheski et al., 2002) and in men (Henderson et al., (2009); Smith et al., 2008). However, previous studies were limited in the number of subjects studied in each age group (young vs. older) and most did not evaluate skeletal muscle breakdown rates and net protein balance, which is important for discerning mechanisms of muscle loss. Measurement of muscle protein synthesis without the characterization of muscle protein breakdown reflects changes in protein turnover, not necessarily protein anabolism. Further, the high variability in the measure of muscle protein synthesis (fractional synthetic rate; FSR) reflects the potential insensitivity of this measure when utilizing it as the sole criteria for determining body protein kinetics and interventional effects (Mittendorfer et al., 2005). The variability in muscle protein FSR is further exacerbated when comparing values between laboratories (Mittendorfer et al., 2005). These issues can make it difficult to distinguish small, but potentially significant differences in basal rates, as is likely with age‐related loss of body protein (Symons et al., 2007).

Consideration of kinetics at the whole‐body level is limited in previous literature comparing age‐related protein kinetics (Henderson et al., 2009). Evaluation of protein kinetics at the whole‐body level not only accounts for changes in muscle protein (~24%–36%), but changes in organ protein and splanchnic utilization as well (Deutz & Wolfe, 2013; Nair et al., 1988). Thus, the examination of whole‐body kinetics, in conjunction with muscle, offers insight into three of the four important kinetic variables required to determine body protein status: 1) whole‐body protein synthesis, 2) whole‐body protein breakdown, and 3) muscle protein synthesis. Given that the methodology involved with the examination of protein kinetics is rather expensive, the sample size required to discern a difference in basal kinetics is also not often feasible. Therefore, in order to more comprehensively examine potential differences in basal protein kinetics between young and older men and women, this study combined and examined data from eight different metabolic studies performed in the same laboratory, utilizing the same tracer methodology.

## 2 METHODS

### 2.1 Subjects

Basal protein kinetics from 91 younger (18–38 years; 53% female) and 55 older (51–81 years; 53% female) adults were combined for analysis (Table 1). Subjects were healthy, with no active malignancies, diabetes, chronic inflammatory disease or other chronic diseases/unstable medical condition, history of gastric bypass surgery, low hematocrit or hemoglobin concentrations, or using corticosteroids; women were not pregnant. All individuals were participating in <2d/wk of resistance exercise. All participants provided written informed consent and protocols were approved by the Institutional Review Board at the University of Arkansas for Medical Sciences.

**Table 1:** Subject characteristics (Mean ± *SD*)

	Young	Older
*N*	91	55
Age (yrs)	25.1 ± 5.5	65.7 ± 7.3
Height (cm)	172.3 ± 9.2	172.0 ± 9.1
Weight (kg)*	74.5 ± 14.2	81.5 ± 11.2
%BF*	27.6 ± 8.3	34.6 ± 6.8
LBM (kg)	48.4 ± 10.6	49.5 ± 9.5

	Young Male	Older Male
*N*	44	26
Age (yrs)	25.7 ± 5.3	65.5 ± 7.5
Height (cm)	177.6 ± 6.5	179.8 ± 5.3
Weight (kg)	81.5 ± 11.9	89.7 ± 8.2
%BF	23.5 ± 6.8	29.6 ± 5.9
LBM (kg)	56.3 ± 7.0	58.1 ± 5.7

	Young Female	Older Female
*N*	47	29
Age (yrs)	24.7 ± 5.7	65.9 ± 7.3
Height (cm)	167.3 ± 8.6	165.0 ± 5.4
Weight (kg)	67.9 ± 13.1	74.2 ± 7.9
%BF	31.5 ± 7.8	39.0 ± 4.0
LBM (kg)	40.9 ± 7.6	41.9 ± 4.0

Note

%BF: percent body fat; LBM: lean body mass.

* Significant difference between young and old (*p* < .05).

### 2.2 Experimental design

Measures of basal protein kinetics from eight different studies collected over five years were combined for analysis. Five of the studies included older individuals (Kim, et al., 2018; Kim et al., 2015; Kim, et al., 2018; Park, et al., 2020)(IRB#204291); three of the studies included young individuals (Kim et al., 2016; Park, et al., 2020)(#205366). All studies had equal or near equal representation of males and females. All participants underwent a metabolic study to determine whole‐body protein kinetics and muscle FSR using a primed continuous infusion of stable isotope tracers. Plasma essential amino acid concentrations were also measured. Body composition was determined from a dual‐energy X‐ray absorptiometry whole‐body scan (QDR‐4500A; Hologic, Waltham, MA). Prior to the metabolic study, all participants were instructed to arrive at the lab following an overnight fast, beginning at 2200hrs the night before, and refrain from strenuous physical activity >72 hr prior. In five of the studies, accounting for 53% of the older and 82% of the young cohorts, participants consumed a run‐in diet for 2–3 days prior to the metabolic study. Diets were based on habitual dietary intake, designed to maintain weight and provide 10%–15% calories from protein.

### 2.3 Stable isotope tracer infusion

In vivo whole‐body protein kinetics was determined via primed continuous infusions of L‐[ring‐^2^H_5_]phenylalanine (prime: 3.92 µmol/kg; infusion rate: 4.60 µmol kg^‐1^ h^‐1^) and L‐[ring‐^2^H_2_]tyrosine (prime: 1.57 µmol/kg; infusion rate: 0.95 µmol kg^‐1^ h^‐1^). A catheter was placed into each arm; one for infusion of stable isotope tracer and the other for the sampling of arterialized blood via the heated hand method (Abumrad et al., 1981). Prior to tracer infusion, a baseline blood sample was collected for the determination of background isotope enrichments. To achieve isotopic equilibrium of L‐[ring‐^2^H_4_]‐tyrosine enrichment derived from L‐[ring‐^2^H_5_]phenylalanine tracer infused, a priming dose of L‐[ring‐^2^H_4_]tyrosine was also given (prime: 0.33 µmol/kg). Blood samples were taken throughout the basal steady‐state (Mean [Range]: 180min [120–270min]) to determine tracer enrichment and plasma essential amino acids. Muscle biopsies from the vastus lateralis were obtained in the basal state for the determination of muscle protein FSR; average time between biopsies was 120min (Range: 90–210min). All procedures were performed by the same research personnel for all studies. Isotope tracers were purchased from Cambridge Isotope Laboratories (Andover, MA). All metabolic studies were completed at the Reynolds Institute on Aging at the University of Arkansas for Medical Sciences.

### 2.4 Analytic methods

Plasma and muscle isotopic tracer enrichment were determined by gas chromatography‐mass spectrometry (GC‐MS; Models 7890A/5975; Agilent Technologies, Santa Clara, CA). Essential amino acid concentrations were measured by liquid chromatography‐mass spectrometry (LC‐MS; QTRAP 5,500 MS; AB SCIEX, Foster City, CA) utilizing the internal standard technique as previously described. (Kim et al., 2016; Kim, et al., 2018).

### 2.5 Calculations

Whole‐body protein kinetics were calculated based on the rate of appearance (R_a_) of isotope tracers, since the rate of appearance and disappearance is equal during the basal state (Equations (1), (2), (3), (4), (5), (6)). Protein synthesis (PS) was calculated as the difference between R_a_ of phenylalanine (Phe) and the rate of hydroxylation of Phe to tyrosine (Tyr) (Equation 4). Protein breakdown (PB) was determined from the R_a_ of Phe (Equation 5). Net balance (NB) was calculated as the difference between PS and PB (Equation 6).

(1) $$\text{Total rate of appearance into plasma}\left(R_{a}\right)=F/E$$

(2) $${\text{Fractional R}}_{a}\;\text{of Tyr from Phe}=E_{\text{Tyr M}+4}/E_{\text{Phe M}+5}$$

(3) $$\text{HydX}={\text{Fractional R}}_{a}\;\text{of Tyr from Phe}\times R_{a}\text{Tyr}$$

(4) $$\text{PS}=\left[\left(R_{a}\text{Phe}‐\text{Phe hydroxylation rate}\right)/0.04\right]$$

(5) $$\text{PB}=\left[R_{a}\text{Phe}\times 25\right]$$

(6) NB=PS‐PB

F is the rate of infusion into the venous side. Enrichment (E) is expressed as tracer to tracee ratio (TTR; for PB) or mole percent excess (MPE = TTR/(1 + TTR)); for PS). E_Tyr M+4_ and E_Phe M+5_ are plasma enrichments of L‐[ring‐^2^H_4_]tyrosine and L‐[ring‐^2^H_5_]phenylalanine relative to the basal atomic mass. HydX is the rate of appearance of Tyr derived from Phe via hydroxylation. To convert amino acid to protein, the conversion factor of 0.04 is based upon the assumption that the contribution of Phe to protein is 4% (Biolo et al., 1995).

Muscle protein FSR was determined using the precursor‐product method, (Baumann et al., 1994) calculated using the plateau in Phe enrichment in the basal state (Equation 7). Plasma enrichment was used as the precursor for better integration of whole‐body and muscle rates; however, calculation of FSR was also performed with intracellular enrichment as the precursor.

(7) $$\text{FSR}=\left(\Delta E_{p}/\left(E_{m}\times t\right)\right)\times 60\times 100$$

ΔE_p_ represents the difference in enrichments of bound L‐[ring‐^2^H_5_]phenylalanine in the first and second biopsies. E_m_ is the calculated mean value of the enrichments of L‐[ring‐^2^H_5_]phenylalanine in the plasma pool. T is the time (min) between biopsies. To express FSR in percent per hour, factors of 60 and 100 were used.

### 2.6 Statistical analysis

Prior to analysis, data were examined for potential outliers. One older individual was found to have an FSR > 3 standard deviations from the mean and removed from the analysis. Differences in basal protein kinetics (PS, PB, NB, FSR), EAA concentrations, and hydroxylation between young and older men and women were evaluated using separate two‐way (age × sex) ANOVAs. Significant interaction and main effects were evaluated using pairwise comparisons with Bonferroni corrections for multiple comparisons and independent samples *t* tests, respectively. FSR was not measured in one of the previous studies with older adults (Kim et al., 2015); EAA concentrations were not measured in two of the previous studies with older adults (Kim, et al., 2018; Kim et al., 2015). Thus, FSR and EAA were evaluated in a subsample of 45 (51% female) and 32 older adults (50% female), respectively. All analyses were performed using SPSS (Version 26, IBM, Armonk, NY, USA), using an α = 0.05 to determine statistical significance.

## 3 RESULTS

There were no group × sex interaction effect for whole‐body protein kinetics (*p* > .05). Relative to LBM, older individuals had greater basal PS (mean difference old‐young (Δ) ± SE: 28.54 ± 8.15 mg/kg LBM/hr; *p* = .001) and PB (Δ: 15.44 ± 7.33 mg/kgLBM/hr; *p* = .038), but a less negative NB (Mean ± *SD*: Young: −31.22 ± 7.42 mg/kg LBM/hr; Old: −18.11 ± 21.60 mg/kg LBM/hr; *p* < .001) compared to young individuals (Table 2). Females had greater basal PS (Δ: 19.10 ± 7.00 mg/kgLBM/hr; *p* = .007) and PB (Δ: 19.22 ± 6.31 mg/kgLBM/hr; *p* = .003) compared to males, but there was no difference in NB (Males: −26.22 ± 14.32 mg/kgLBM/hr; Females: −26.34 ± 17.09 mg/kgLBM/hr).

**Table 2:** Basal protein kinetics (Mean ± *SD*): updated

	Full Group		Male		Female	
	Young	Older	Young	Older	Young	Older
PS (mg/kg LBM/hr)*^#^	206.39 ± 28.13	234.93 ± 56.31	195.61 ± 24.50	226.80 ± 66.37	216.48 ± 27.79	242.22 ± 45.46
PB (mg/kg LBM/hr)*^#^	237.61 ± 30.80	253.05 ± 48.83	226.66 ± 5.67	244.86 ± 7.37	247.86 ± 5.48	260.39 ± 6.98
NB (mg/kg LBM/hr)*	−31.22 ± 7.42	−18.11 ± 21.6	−31.05 ± 7.86	−18.06 ± 18.68	−31.38 ± 7.06	−18.17 ± 24.26
FSR (%/hr)^#^	0.049 ± 0.014	0.056 ± 0.023	0.045 ± 0.012	0.053 ± 0.022	0.053 ± 0.014	0.059 ± 0.024
[EAA] (µmol/min)^β^	834.98 ± 96.81	830.30 ± 105.22	885.41 ± 72.53	838.95 ± 100.04	787.76 ± 93.32	821.64 ± 112.75
Hydroxylation (µmol/min)*	0.150 ± 0.027	0.114 ± 0.047	0.156 ± 0.027	0.116 ± 0.045	0.145 ± 0.026	0.112 ± 0.049

Note

PS: protein synthesis; PB: protein breakdown; NB: net balance; FSR: fractional synthetic rate; Significant difference between *young and old, ^#^male and female, ^β^young male and female (*p* < .05).

There were no group × sex interaction effect for FSR (*p* = .739; *p* = .575). When calculated with plasma enrichment as the precursor, basal FSR was not significantly different between young and older (Δ: 0.007 ± 0.003%/hr; *p* = .052), but was higher in females compared to males (Δ: 0.008 ± 0.003%/hr; *p* = .012). When calculated with intracellular enrichment as the precursor, there was not a significant difference between young and old (Δ: 0.003 ± 0.004%/hr; *p* = .370), or between males and females (Δ: 0.007 ± 0.004%/hr; *p* = .075).

There was a group × sex interaction for [EAA] (*p* = .032), with young males having greater [EAA] than young females (Δ: 97.65 ± 18.92 µmol/L; *p* < .001), but no differences between older males and females (Δ: 17.31 ± 31.88 µmol/L; *p* = .588), young and older men (Δ: 46.46 ± 26.33 µmol/L; *p* = .080) or young and older women (Δ: 33.88 ± 26.10 µmol/L; *p* = .197) (Table 2). Relative to LBM, females had greater [EAA] than males (Δ: 4.17 ± 0.51 µmol/L/kgLBM; *p* < .001). For hydroxylation, there was no interaction effect (*p* = .628). Hydroxylation was significantly higher in young compared to older (Δ: 0.036 ± 0.007 µmol/min; *p* < .001; Table 2); there was no difference between males and females (Δ: 0.008 ± 0.007 µmol/min; *p* = .200).

## 4 DISCUSSION

Previous research examining differences in basal protein kinetics between young and older individuals has been limited by a lack of evaluation of whole‐body protein breakdown rates and net balance. Results of the current study show that in the basal state, older individuals have greater whole‐body PS and PB and a less negative NB compared to younger individuals, but no differences in muscle protein synthesis. Women were also found to have higher protein turnover than men, but no difference in net balance. These data demonstrate a difference in fasted protein kinetics between young and older adults, with a potential influence of sex that does not explain age‐related loss of body protein.

Previous research on basal protein kinetics has focused on differences in muscle protein synthesis, with most studies showing no significant differences between young and older individuals (Cuthbertson et al., 2005; Kumar et al., 2009; Markofski et al., 2015; Moore et al., 2015; Volpi et al., 2001). The present study uniquely evaluated whole‐body protein kinetics, which not only allows for the consideration of PB and NB, in addition to PS, but also allows for the consideration of the whole‐body protein pool. In the fasted state, muscle protein synthesis contributes about ~24%–36% to whole‐body protein turnover (Nair et al., 1988). Contribution of muscle to whole‐body catabolism has been shown to be reduced in healthy, older adults (Morais et al., 1997), making consideration of the whole‐body protein pool important when evaluating aging populations, especially in the fasted state. More importantly, the combined measures provide a better representation of body protein status/balance. In the current study, higher PS and PB observed in older individuals reflect a greater basal turnover in whole‐body protein. Greater protein turnover may be reflective of a greater need for the replacement of less effective/efficient proteins in older individuals (Fitts et al., 2007). The less negative NB reflects a more efficient reutilization of the existing amino acid pool, oxidizing fewer amino acids compared to young individuals. This idea is generally supported by the lower hydroxylation rates observed in the older group. When evaluating synthesis and breakdown rates across the leg, Volpi et al. (2001) found older adults to have greater synthesis and breakdown rates compared to young adults. Net balance was also found to be similar between young and older adults; however, the authors pointed out that their findings were underpowered and that a large number of subjects (100+) would be needed to observe a significant age‐related difference in basal muscle protein turnover in a cross‐sectional study (Volpi et al., 2001). Although the current study did not evaluate MPB, the observed elevation in whole‐body PS and PB in this study is consistent with the conclusion of Volpi et al. (2001) of greater protein turnover in older subjects. Finally, differences in whole‐body kinetics with minimal differences in muscle protein kinetics may reflect age‐related metabolic alterations centered on the splanchnic bed, rather than skeletal muscle, in the fasted state (Volpi et al., 1999).

Previous research has consistently reported no significant difference in FSR between young and older individuals (Cuthbertson et al., 2005; Kumar et al., 2009; Markofski et al., 2015; Moore et al., 2015; Volpi et al., 2001), but potentially higher in older women compared to older men (Henderson et al., 2009; Smith et al., 2008). The consolidation of our data is consistent with these findings, showing minimal differences in FSR between young and older adults, but potentially slightly higher FSR in women compared to men, regardless of age. Measurements of muscle protein metabolism are highly variable, between and within studies and individuals (Smith et al., 2011). Basal values range from 0.021% to 0.055% for young to middle‐aged individuals, making it difficult to distinguish differences. Previous studies have reported a slightly higher FSR in older compared to young individuals (Δ: 0.002%–0.004%/hr) and in women (Δ: 0.003%–0.004%/hr)( Henderson et al., 2009), despite nonsignificant statistical differences (Kumar et al., 2009; Markofski et al., 2015; Volpi et al., 2001), with an equal number showing slightly lower FSR in older (Δ: 0.001%–0.004%/hr) (Cuthbertson et al., 2005; Henderson et al., 2009; Moore et al., 2015). Of greater importance, few studies, including the current study, have examined both MPS and MPB simultaneously. Without the evaluation of both variables, conclusions about anabolism are difficult to make. In the fasted state, muscle serves as an amino acid reservoir to maintain protein balance in essential organs and plasma amino acid levels. Differences between young and older at the whole‐body level, but not at the muscle level, suggest differences in nonmuscle protein metabolism. This emphasizes the importance of accounting for nonmuscle protein requirements when considering strategies to maintain muscle in aging individuals.

In contrast to the results of the current study, Henderson et al. (2009) found whole‐body protein kinetics and muscle protein synthesis to be lower in older adults compared to young. Explanations for the contrasting results are not immediately clear, but may be related to modifiable lifestyle factors, such as diet and exercise, in addition to high population variability. The implementation of run‐in diets, or the lack thereof, has been suggested to potentially influence protein kinetics (Henderson et al., 2009). Run‐in diets were used in a majority, but not all of the studies included in the current analysis, which may have introduced some level of variability. However, Gorissen et al. (2017) found no effect of 14 days of protein habituation on FSR in the basal period (Gorissen et al., 2017), suggesting that a 3‐day run‐in diet would have minimal impact. The run‐in diets were also designed to provide protein intakes similar to habitual intake (10%–15%); therefore, the implementation or lack thereof, likely had a minimal impact on basal kinetics. Habitual protein intake may also have an impact on protein kinetics. Recent results from Højfeldt et al. (2020) show that three weeks of habituation to a high protein diet (>2.1 g/kgLM/d) altered basal, whole‐body protein kinetics in older men, resulting in a more negative fasted net balance that resembled that of young individuals (Højfeldt et al., 2020). Although habitual protein intake was not evaluated in the current study, population‐level data indicate that, on average, older individuals consume less dietary protein than young individuals (88–87g vs. 81–71g or 1.3 g/kg vs. 1.0 g/kg) (Berryman et al., 2018; Traylor et al., 2018). In the context of the current study, this would amount to ~ 97g of protein for the average young individual and ~ 81g of protein for the average older individual, a difference that has been shown to impact muscle protein synthesis in older adults (Dillon et al., 2009). These data, in combination with those of the current study, support the notion that the basis for age‐related loss of body protein is related to the inefficient handling of dietary protein, not changes in basal, protein kinetics or impaired synthetic capacity. Inefficient handling of dietary protein may be due to a number of factors, including impaired protein digestion and amino acid absorption, and greater first‐pass splanchnic uptake with aging (Boirie et al., 1997; Burd et al., 2013; Volpi et al., 1999). All of these changes would translate to decreased postprandial availability of exogenous amino acids with aging. In contrast, higher protein intakes are often associated with greater protein turnover. Future research with detailed characterization or control of dietary intake is needed to elucidate this theory. Finally, of significance, inter‐individual variability in whole‐body kinetics is likely very high. Results from Henderson et al. fall within the variability (1 *SD*) of the current study, suggesting that data from both studies may reflect high population variability, especially in older adults. Future studies that control or account for lifestyle factors are needed to establish these differences. Despite differences between the current study and those of Henderson et al., both studies did find greater turnover and potentially higher FSR in women compared to men, regardless of age. Reason for sex differences in protein and muscle kinetics have not been elucidated, but are commonly attributed to the effects of sex hormones, with estrogen suggested to have protective effects (Hansen, 2018; Smith et al., 2008). However, the presence of differences between men and women, both at pre and postmenopausal stages suggests other factors beyond hormones, such as lifestyle or genetics, may also have an effect (Henderson et al., 2009). Women have been shown to consume less protein on average than men, both at young (70g vs. 110g) and older ages (69g vs. 92g) (Berryman et al., 2018). Although there was no difference in net balance, higher protein turnover in women may reflect a similar mechanism of protein recycling observed in older adults, but further research is needed to understand sex‐related differences in protein kinetics, especially in the context of aging.

It is important to consider that the current study focuses on kinetic differences in the basal state. The imbalance between PS and PB in the fasted state is small, making it difficult to fully elucidate the impact of these differences over time. The fasted state also only represents approximately 1/3 of the daily kinetic response, whereas the repeated kinetic responses following nutrient/protein ingestion are likely have a greater impact on overall protein balance over time. These results do suggest that basal whole‐body protein kinetics may change with age and sex. These changes may be a compensatory mechanism for less‐optimal protein intake and/or a reduced efficiency in nonmuscle protein metabolic processes, such as digestion and absorption kinetics in older individuals requiring greater retention and more efficient utilization of amino acids. Further research is needed to confirm this theory. The considerable research demonstrating anabolic‐resistance in older individuals indicates a reduced protein intake, digestion, and utilization of protein compared to young individuals. Therefore, given the realities of differences in nutritional intake and physiological efficiency, it would be advantageous to alter protein turnover to utilize endogenous amino acids as efficiently as possible. This is consistent with the requirement for higher protein intake to maximally stimulate MPS (Burd et al., 2013; Moore et al., 2015) and greater first‐pass splanchnic uptake in older adults (Volpi et al., 1999). Further research into these mechanisms may be insightful for continuing to understand the mechanisms associated with age‐related muscle loss.

## CONFLICT OF INTEREST

All authors report no conflict of interest.

## AUTHOR CONTRIBUTIONS

KRH, DDC, AAF conceived the idea; IYK, SP, RRW, AAF planned the original experiments; IYK, SP performed the original experiments; KRH compiled the results and completed analyses; KRH lead in writing the manuscript; All authors (KRH, DDC, IYK, SP, RRW, AAF) provided critical feedback that helped shape the research analysis and manuscript.

## ETHICAL STATEMENT

All procedures reported upon were conducted in accordance with the ethical standards of the responsible committee on human experimentation (institutional and national) and with the Helsinki Declaration of 1975, as revised in 2008.

## Data Availability

Data are available upon reasonable request.

## References

[phy214633-bib-0001] Abumrad, N. N. , Rabin, D. , Diamond, M. P. , & Lacy, W. W. (1981). Use of a heated superficial hand vein as an alternative site for the measurement of amino acid concentrations and for the study of glucose and alanine kinetics in man. Metabolism, 30, 936–940. 10.1016/0026-0495(81)90074-3 7022111

[phy214633-bib-0002] Balagopal, P. , Rooyackers, O. E. , Adey, D. B. , Ades, P. A. , & Nair, K. S. (1997). Effects of aging on in vivo synthesis of skeletal muscle myosin heavy‐chain and sarcoplasmic protein in humans. American Journal of Physiology‐Endocrinology and Metabolism, 273, E790–E800. 10.1152/ajpendo.1997.273.4.e790 9357810

[phy214633-bib-0003] Baumann, P. Q. , Stirewalt, W. S. , O'Rourke, B. D. , Howard, D. , & Nair, K. S. (1994). Precursor pools of protein synthesis: A stable isotope study in a swine model. American Journal of Physiology, 267, E203–E209. 10.1152/ajpendo.1994.267.2.e203 8074199

[phy214633-bib-0004] Berryman, C. E. , Lieberman, H. R. , Fulgoni, V. L. 3rd , & Pasiakos, S. M. (2018). Protein intake trends and conformity with the Dietary Reference Intakes in the United States: Analysis of the National Health and Nutrition Examination Survey, 2001–2014. American Journal of Clinical Nutrition, 108, 405–413. 10.1093/ajcn/nqy088 29931213

[phy214633-bib-0005] Biolo, G. , Declan Fleming, R. Y. , & Wolfe, R. R. (1995). Physiologic hyperinsulinemia stimulates protein synthesis and enhances transport of selected amino acids in human skeletal muscle. Journal of Clinical Investigation, 95, 811–819. 10.1172/jci117731 7860765 PMC295560

[phy214633-bib-0006] Boirie, Y. , Gachon, P. , & Beaufrere, B. (1997). Splanchnic and whole‐body leucine kinetics in young and elderly men. The American Journal of Clinical Nutrition, 65, 489–495. 10.1093/ajcn/65.2.489 9022534

[phy214633-bib-0007] Burd, N. A. , Gorissen, S. H. , & van Loon, L. J. (2013). Anabolic resistance of muscle protein synthesis with aging. Exercise and Sport Sciences Reviews, 41, 169–173. 23558692 10.1097/JES.0b013e318292f3d5

[phy214633-bib-0008] Cuthbertson, D. , Smith, K. , Babraj, J. , Leese, G. , Waddell, T. , Atherton, P. , …, Rennie, M. J. (2005). Anabolic signaling deficits underlie amino acid resistance of wasting, aging muscle. The FASEB Journal, 19, 422–424. 15596483 10.1096/fj.04-2640fje

[phy214633-bib-0009] Deutz, N. E. , & Wolfe, R. R. (2013). Is there a maximal anabolic response to protein intake with a meal? Clinical Nutrition, 32, 309–313. 23260197 10.1016/j.clnu.2012.11.018 PMC3595342

[phy214633-bib-0010] Dillon, E. L. , Sheffield‐Moore, M. , Paddon‐Jones, D. , Gilkison, C. , Sanford, A. P. , Casperson, S. L. , …, Urban, R. J. (2009). Amino acid supplementation increases lean body mass, basal muscle protein synthesis, and insulin‐like growth factor‐I expression in older women. Journal of Clinical Endocrinology and Metabolism, 94, 1630–1637. 19208731 10.1210/jc.2008-1564 PMC2684480

[phy214633-bib-0011] Doherty, T. J. (2001). The influence of aging and sex on skeletal muscle mass and strength. Current Opinion in Clinical Nutrition and Metabolic Care, 4, 503–508. 11706284 10.1097/00075197-200111000-00007

[phy214633-bib-0012] Fitts, R. H. , Romatowski, J. G. , Peters, J. R. , Paddon‐Jones, D. , Wolfe, R. R. , & Ferrando, A. A. (2007). The deleterious effects of bed rest on human skeletal muscle fibers are exacerbated by hypercortisolemia and ameliorated by dietary supplementation. American Journal of Physiology Cell Physiology, 293, C313–C320. 17409123 10.1152/ajpcell.00573.2006

[phy214633-bib-0013] Gorissen, S. H. , Horstman, A. M. , Franssen, R. , Kouw, I. W. , Wall, B. T. , Burd, N. A. , …, van Loon, L. J. (2017). Habituation to low or high protein intake does not modulate basal or postprandial muscle protein synthesis rates: A randomized trial. American Journal of Clinical Nutrition, 105, 332–342. 10.3945/ajcn.115.129924 27903518

[phy214633-bib-0014] Hansen, M. (2018). Female hormones: Do they influence muscle and tendon protein metabolism? The Proceedings of the Nutrition Society, 77, 32–41. 28847313 10.1017/S0029665117001951

[phy214633-bib-0015] Henderson, G. C. , Dhatariya, K. , Ford, G. C. , Klaus, K. A. , Basu, R. , Rizza, R. A. , …, Nair, K. S. (2009). Higher muscle protein synthesis in women than men across the lifespan, and failure of androgen administration to amend age‐related decrements. The FASEB Journal, 23, 631–641. 18827019 10.1096/fj.08-117200 PMC2630787

[phy214633-bib-0016] Højfeldt, G. , Bülow, J. , Agergaard, J. , Asmar, A. , Schjerling, P. , Simonsen, L. , …, Holm, L. (2020). Impact of habituated dietary protein intake on fasting and postprandial whole‐body protein turnover and splanchnic amino acid metabolism in elderly men: A randomized, controlled, crossover trial. American Journal of Clinical Nutrition. 10.1093/ajcn/nqaa201 32710741

[phy214633-bib-0017] Janssen, I. , Heymsfield, S. B. , Wang, Z. M. , & Ross, R. (2000). Skeletal muscle mass and distribution in 468 men and women aged 18–88 yr. Journal of Applied Physiology (1985), 89, 81–88. 10.1152/jappl.2000.89.1.81 10904038

[phy214633-bib-0018] Kim, I. Y. , Schutzler, S. , Schrader, A. , Spencer, H. J. , Azhar, G. , Ferrando, A. A. , & Wolfe, R. R. (2016). The anabolic response to a meal containing different amounts of protein is not limited by the maximal stimulation of protein synthesis in healthy young adults. American Journal of Physiology. Endocrinology and Metabolism, 310, E73–E80. 26530155 10.1152/ajpendo.00365.2015 PMC4675798

[phy214633-bib-0019] Kim, I. Y. , Schutzler, S. , Schrader, A. M. , Spencer, H. J. , Azhar, G. , Wolfe, R. R. , & Ferrando, A. A. (2018). Protein intake distribution pattern does not affect anabolic response, lean body mass, muscle strength or function over 8 weeks in older adults: A randomized‐controlled trial. Clinical Nutrition, 37, 488–493. 28318687 10.1016/j.clnu.2017.02.020 PMC9252263

[phy214633-bib-0020] Kim, I.‐Y. , Schutzler, S. , Schrader, A. , Spencer, H. , Kortebein, P. , Deutz, N. E. , …, Ferrando, A. A. (2015). Quantity of dietary protein intake, but not pattern of intake, affects net protein balance primarily through differences in protein synthesis in older adults. American Journal of Physiology‐Endocrinology and Metabolism, 308, E21–E28. 25352437 10.1152/ajpendo.00382.2014 PMC4280213

[phy214633-bib-0021] Kim, I. Y. , Shin, Y. A. , Schutzler, S. E. , Azhar, G. , Wolfe, R. R. , & Ferrando, A. A. (2018). Quality of meal protein determines anabolic response in older adults. Clinical Nutrition, 37, 2076–2083. 29066101 10.1016/j.clnu.2017.09.025 PMC9987475

[phy214633-bib-0022] Kumar, V. , Selby, A. , Rankin, D. , Patel, R. , Atherton, P. , Hildebrandt, W. , …, Rennie, M. J. (2009). Age‐related differences in the dose‐response relationship of muscle protein synthesis to resistance exercise in young and old men. Journal of Physiology, 587, 211–217. 10.1113/jphysiol.2008.164483 19001042 PMC2670034

[phy214633-bib-0023] Markofski, M. M. , Dickinson, J. M. , Drummond, M. J. , Fry, C. S. , Fujita, S. , Gundermann, D. M. , …, Volpi, E. (2015). Effect of age on basal muscle protein synthesis and mTORC1 signaling in a large cohort of young and older men and women. Experimental Gerontology, 65, 1–7. 10.1016/j.exger.2015.02.015 25735236 PMC4397165

[phy214633-bib-0024] Mitchell, W. K. , Atherton, P. J. , Williams, J. , Larvin, M. , Lund, J. N. , & Narici, M. (2012). Sarcopenia, dynapenia, and the impact of advancing age on human skeletal muscle size and strength; a quantitative review. Frontiers in Physiology, 3, 260 10.3389/fphys.2012.00260 22934016 PMC3429036

[phy214633-bib-0025] Mittendorfer, B. , Andersen, J. , Plomgaard, P. , Saltin, B. , Babraj, J. , Smith, K. , & Rennie, M. (2005). Protein synthesis rates in human muscles: Neither anatomical location nor fibre‐type composition are major determinants. The Journal of Physiology, 563, 203–211. 10.1113/jphysiol.2004.077180 15611031 PMC1665563

[phy214633-bib-0026] Moore, D. R. , Churchward‐Venne, T. A. , Witard, O. , Breen, L. , Burd, N. A. , Tipton, K. D. , & Phillips, S. M. (2015). Protein ingestion to stimulate myofibrillar protein synthesis requires greater relative protein intakes in healthy older versus younger men. Journals of Gerontology Series A: Biomedical Sciences and Medical Sciences, 70, 57–62. 10.1093/gerona/glu103 25056502

[phy214633-bib-0027] Morais, J. A. , Gougeon, R. , Pencharz, P. B. , Jones, P. , Ross, R. , & Marliss, E. B. (1997). Whole‐body protein turnover in the healthy elderly. The American Journal of Clinical Nutrition, 66, 880–889. 10.1093/ajcn/66.4.880 9322564

[phy214633-bib-0028] Nair, K. S. , Halliday, D. , & Griggs, R. C. (1988). Leucine incorporation into mixed skeletal muscle protein in humans. American Journal of Physiology, 254, E208–E213. 10.1152/ajpendo.1988.254.2.e208 3279803

[phy214633-bib-0029] Park, S. , Church, D. D. , Azhar, G. , Schutzler, S. E. , Ferrando, A. A. , & Wolfe, R. R. (2020). Anabolic response to essential amino acid plus whey protein composition is greater than whey protein alone in young healthy adults. Journal of the International Society of Sports Nutrition, 17, 9. 32041644 10.1186/s12970-020-0340-5 PMC7011510

[phy214633-bib-0030] Park, S. , Church, D. D. , Starck, C. , Schutzler, S. E. , Azhar, G. , Kim, I. Y. , …, Wolfe, R. R. (2020). The impact of Hayward green kiwifruit on dietary protein digestion and protein metabolism. European Journal of Nutrition. 10.1007/s00394-020-02363-5 PMC7900049 32970234

[phy214633-bib-0031] Phillips, S. M. , Glover, E. I. , & Rennie, M. J. (2009). Alterations of protein turnover underlying disuse atrophy in human skeletal muscle. Journal of Applied Physiology, 107, 645–654. 10.1152/japplphysiol.00452.2009 19608931

[phy214633-bib-0032] Smith, G. I. , Atherton, P. , Villareal, D. T. , Frimel, T. N. , Rankin, D. , Rennie, M. J. , & Mittendorfer, B. (2008). Differences in muscle protein synthesis and anabolic signaling in the postabsorptive state and in response to food in 65–80 year old men and women. PLoS One, 3, e1875 10.1371/journal.pone.0001875 18365020 PMC2267222

[phy214633-bib-0033] Smith, G. I. , Patterson, B. W. , & Mittendorfer, B. (2011). Human muscle protein turnover—why is it so variable? Journal of Applied Physiology, 110, 480–491. 10.1152/japplphysiol.00125.2010 21109595 PMC3043790

[phy214633-bib-0034] Symons, T. B. , Schutzler, S. E. , Cocke, T. L. , Chinkes, D. L. , Wolfe, R. R. , & Paddon‐Jones, D. (2007). Aging does not impair the anabolic response to a protein‐rich meal. American Journal of Clinical Nutrition, 86, 451–456. 10.1093/ajcn/86.2.451 17684218

[phy214633-bib-0035] Tieland, M. , Trouwborst, I. , & Clark, B. C. (2018). Skeletal muscle performance and ageing. J Cachexia Sarcopenia Muscle, 9, 3–19. 29151281 10.1002/jcsm.12238 PMC5803609

[phy214633-bib-0036] Traylor, D. A. , Gorissen, S. H. M. , & Phillips, S. M. (2018). Perspective: Protein requirements and optimal intakes in aging: Are we ready to recommend more than the recommended daily allowance? Advances in Nutrition, 9, 171–182. 10.1093/advances/nmy003 29635313 PMC5952928

[phy214633-bib-0037] Volpi, E. , Mittendorfer, B. , Wolf, S. E. , & Wolfe, R. R. (1999). Oral amino acids stimulate muscle protein anabolism in the elderly despite higher first‐pass splanchnic extraction. American Journal of Physiology‐Endocrinology and Metabolism. 277, E513–E520. 10.1152/ajpendo.1999.277.3.e513 10484364

[phy214633-bib-0038] Volpi, E. , Sheffield‐Moore, M. , Rasmussen, B. B. , & Wolfe, R. R. (2001). Basal muscle amino acid kinetics and protein synthesis in healthy young and older men. JAMA, 286, 1206–1212. 10.1001/jama.286.10.1206 11559266 PMC3183815

[phy214633-bib-0039] Welle, S. , Thornton, C. , Jozefowicz, R. , & Statt, M. (1993). Myofibrillar protein synthesis in young and old men. American Journal of Physiology‐Endocrinology and Metabolism, 264, E693–E698. 10.1152/ajpendo.1993.264.5.e693 8498491

[phy214633-bib-0040] Wolfe, R. R. (2006). The underappreciated role of muscle in health and disease. American Journal of Clinical Nutrition, 84, 475–482. 10.1093/ajcn/84.3.475 16960159

[phy214633-bib-0041] Yarasheski, K. E. , Welle, S. , & Nair, K. S. (2002). Muscle protein synthesis in younger and older men. JAMA, 287, 317–318. 10.1001/jama.287.3.317 11790208

